# Synergistic Effects of *Limosilactobacillus fermentum* ASBT-2 with Oxyresveratrol Isolated from Coconut Shell Waste

**DOI:** 10.3390/foods10112548

**Published:** 2021-10-22

**Authors:** Vidhya Prakash, Akshaya S Krishnan, Reshma Ramesh, Chinchu Bose, Girinath G. Pillai, Bipin G. Nair, Sanjay Pal

**Affiliations:** 1 School of Biotechnology, Amrita Vishwa Vidyapeetham, Kollam 690525, India; vidhyamc@gmail.com (V.P.); akshayaskrishnan7@gmail.com (A.S.K.); reshmaramesh2203@gmail.com (R.R.); chinchubose@gmail.com (C.B.); bipinnair03@gmail.com (B.G.N.); 2 Discovery Chemistry, Nyro Research India, Kochi 682021, India; pillai@nyroindia.org

**Keywords:** polyphenols, oxyresveratrol, bioactivity, probiotics, value addition, synbiotic, MIC, antagonism, molecular docking

## Abstract

Value-added phytochemicals from food by-products and waste materials have gained much interest and among them, dietary polyphenolic compounds with potential biological properties extend a promising sustainable approach. Oxyresveratrol (Oxy), a stilbenoid polyphenol, possesses great therapeutic potential, though its pharmacokinetic issues need attention. A good source of oxyresveratrol was found in underutilized coconut shells and the synbiotic applications of the compound in combination with a potential probiotic isolate *Limosilactobacillus fermentum* ASBT-2 was investigated. The compound showed lower inhibitory effects on the strain with minimum inhibitory concentration (MIC) of 1000 µg/mL. Oxyresveratrol at sub-MIC concentrations (500 µg/mL and 250 µg/mL) enhanced the probiotic properties without exerting any inhibitory effects on the strain. The combination at sub- MIC concentration of the compound inhibited *Salmonella enterica* and in silico approaches were employed to elucidate the possible mode of action of oxy on the pathogen. Thus, the combination could target pathogens in the gut without exerting negative impacts on growth of beneficial strains. This approach could be a novel perspective to address the poor pharmacokinetic properties of the compound.

## 1. Introduction

Naturally occurring compounds have a wide range of acceptability for the prevention of disease and health promotion and thus they continue to attract worldwide attention. There is extensive research across the globe investigating the nutritional and health benefits of a large number of natural compounds [1]. Among these natural products, polyphenols have been extensively studied for their antioxidative and anti-inflammatory properties [2]. They are distinguished by the presence of one or more aromatic rings having multiple hydroxyl groups attached to them and possess the ability to alter the gut microbiota constructively [2,3].

Resveratrol (3, 4′, 5-trihyroxystilbene) is a widely studied stilbene that is commonly found in grape skin, peanuts and red wine. There are several studies indicating the antimicrobial effects of resveratrol against the pathogenic bacteria such as *Salmonella* *typhimurium*, *Staphylococcus aureus*, *Escherichia coli*, *Vibrio cholerae* and *Arcobacter butzleri* [4,5]. In addition, it was shown to enhance the growth of beneficial bacteria belonging to lactic acid bacteria with minimum inhibitory effects on their functional properties [6]. Oxyresveratrol (trans-2,3′,4,5′—tetrahydroxystilbene) is a naturally occurring analog of resveratrol that is found in the heartwood of *Artocarpus lacucha*, *Morus alba* and *Smilax china L*. Though it is structurally similar to resveratrol, the compound exhibits multitudes of pharmacokinetic properties in comparison to resveratrol with many more unexplored modes of action [7].

The coconut obtained from the *Cocos nucifera* plant which belongs to the family *Arecaceae* has wide domestic and industrial demand as a food ingredient and in oil production, which makes it an important agro-product in tropical countries [8,9]. The shells are obtained as by-products from oil industries and are often considered as agro-wastes [9]. Oxyresveratrol (Oxy) was isolated and characterized from coconut shell for the first time in a previously published in-house work which highlighted the role of the underutilized parts of coconut in promoting anticancer effects [10].

Recently, polyphenol–probiotic synergy has been found to establish better modulation of the microbiome composition with positive impacts on their metabolic activities [11,12]. Probiotics have been established for their positive effects on gut immunomodulation, and in addition their interactions with polyphenolic metabolites may play a crucial role in establishing health benefits. Dietary polyphenol metabolism may physiologically alter the microbial populations as they pertain in the gut for longer periods [13,14]. Thus, these combinations could effectively modulate the nutritional strategy valorizing the waste to health approach.

A symbiotic approach was attempted to investigate the effects of sub-MIC concentrations of oxyresveratrol on the probiotic characteristics of a strain *Limosilactobacillus fermentum* ASBT-2 which was isolated and characterized in a previous study [15]. The addition of the compound significantly alleviated probiotic attributes of the strain and demonstrated lesser deleterious effects. Results clearly indicated that addition of the compound promoted the biofilm formation of the strain in simulated intestinal environment, and their significant coaggregation and autoaggregation properties were also retained or improved in the presence of the compound. Inhibition of the enteric strain, *Salmonella enterica*, was further promoted with the combination which led us to attempt in silico docking approaches to elucidate the mode of action of oxyresveratrol against the pathogen.

## 2. Materials and Methods

### 2.1. Raw Materials; Chemicals and Bacterial Strains

Mature coconut shells were procured from Vallikavu, Kollam, Kerala, India. The shells were crushed and coarsely powdered for extraction. Probiotic strain used was *Limosilactobacillus fermentum* ASBT-2. The strain was maintained in De Man, Rogosa, and Sharpe (MRS) broth (HiMedia, Mumbai, India) in 80% *v*/*v* glycerol (HiMedia, Mumbai, India) and subcultured on MRS agar plates. The pathogens used in the study were *Salmonella enterica* MW116733 and *E. coli* MDR (multi-drug resistant). (Clinical strains were gifted by Dr. Bhabatosh Das, THSTI, Faridabad, India). Strains were maintained as stock cultures in Nutrient broth (HiMedia, India) and MRS broth, supplemented with 80% (*v*/*v*) glycerol and stored at −80 °C.

### 2.2. Extraction and Isolation of Oxyresveratrol

Mature coconut shells were crushed, powdered and subjected to soxhlet extraction using petroleum ether, chloroform and ethyl methyl ketone (EMK) (Merck, India). The solvents were removed by rotary flash evaporator at a temperature of 40 °C. Thin layer chromatography (TLC) analysis of the extracts showed oxyresveratrol corresponding to the band specifically in the EMK extract. The EMK extract (1 g) was fractionated using silica gel column chromatography (60–120 mesh). The column elution was initiated using chloroform (Merck, Bengaluru, India) followed by a mixture of chloroform containing increasing proportions of ethyl acetate (Merck, Bengaluru, India). The fractions were monitored by thin layer chromatography (TLC) (toluene: ethyl acetate: formic acid; 5:5:2). Further characterization of the desired compound was carried out using UV Spectrophotometer, HPLC and LC-MS. [10].

#### Characterization of Oxyresveratrol

UV-Vis spectra were recorded on a Shimadzu UV spectrophotometer (Model UV-1800). HPLC was performed on a Shimadzu-SPD-M20A, equipped with DAD (Diode Array detector). The compound was dissolved in HPLC grade methanol (Merck, Bengaluru, India). For routine analysis, a 10–95% gradient elution of acetonitrile (Sigma-Aldrich, India) and Milli-Q water was pumped through the column 40 °C at 1 mL/min over 20 min [16]. Further LC-MS studies were carried out.

### 2.3. Determination of Minimum Inhibitory Concentration (MIC) of Oxyresveratrol

Overnight cultures of *Limosilactobacillus fermentum*-ASBT strain (LF) were adjusted to 0.1 OD_600_ corresponding to McFarland standard of 0.5. An amount of 2 mg oxyresveratrol was diluted in 25 μL dimethyl sulfoxide (DMSO *w*/*v*) (HiMedia, Mumbai, India) and maintained as stock solution (2000 μg/mL). The concentrations of oxyresveratrol used were in the range of 31.25–2000 μg/mL. Untreated bacterial culture (without oxyresveratrol) and bacterial culture treated with DMSO was also maintained. The cultures were incubated at 37 °C for 24 h and MIC was determined by measuring optical density (OD_600_). MIC was considered as the lowest concentration showing visual growth inhibition of the probiotic strain [17].

### 2.4. Effect of Oxyresveratrol on pH Tolerance of the Strain

The pH tolerance of the strain was determined by inoculating the culture at 0.1 OD_600_ in MRS broth maintained at pH 2.0, 3.0, and 5.0 with sub-MIC concentrations of the compound. Briefly, 20 µL of 0.1 OD_600_ adjusted culture was inoculated into 180 µL of MRS broth at different pH and oxyresveratrol at MIC, 1/2 MIC or 1/4 MIC concentrations was added in 96-well plates and incubated at 37 °C. Aliquots of 100 µL were removed at time points 0 and 3 h and plated on MRS agar for determining total viable counts (log CFU/mL). To determine whether oxyresveratrol affects pH tolerance, the viable counts of LF at a specific pH with or without oxyresveratrol was maintained [18,19].

### 2.5. Effect of Oxyresveratrol on Tolerance to Bile Salts

MRS medium with 0.15 and 1.0% (*w*/*v*) bile salts was used (HiMedia, Mumbai, India). A total of 20 µL of 0.1 OD_600_ adjusted culture was inoculated into 180 µL of MRS broth with bile salts and oxyresveratrol at specific MIC concentrations in 96-well plates and incubated at 37 °C. Viable counts after exposure to 3 h (log CFU/mL) were determined [20,21].

### 2.6. Effects on Survival under Simulated Gastrointestinal Conditions

Survival of LF in simulated gastric juice (SGJ) and simulated intestinal juices (SIJ) and to check the efficiency of the strain to form biofilm versus planktonic phenotypes in synergy with oxy was performed as described by Berkes et al. with modifications. OD of the culture was adjusted to 0.1 (OD_600_) and 2 mL of the culture was transferred to 6-well microtiter plate for growing biofilm and planktonic phenotypes. The strains were pelleted, washed in phosphate buffered saline (PBS; pH 7.4) (HiMedia, Mumbai, India) and 1 mL of the cell suspension was resuspended in biorelevant media, fasting state intestinal fluid (FaSSIF), fed state intestinal fluid (FeSSIF), fasting state gastric fluid (FaSSGF) with different concentrations (i.e., MIC, 1/2 MIC or 1/4 MIC) of oxyresveratrol to achieve an (OD_600_) of 0.1. Viable counts were determined after 3 h (log CFU/mL). Fed state and fasting state fluids without compound was maintained as control [22,23].

### 2.7. Effect of Oxyresveratrol on Cell Surface Hydrophobicity of the Strain

Bacterial cells were grown in MRS broth for 24 h at 37 °C, centrifuged (5000× *g*, 15 min, at 4 °C), washed twice and resuspended in PBS (pH 7.4) with different concentrations (MIC, 1/2 MIC, 1/4 MIC) of oxyresveratrol to achieve an OD_600_ of 0.1 (A_0_). n-hexadecane (Merck, Bengaluru, India) was added (1:5 *v*/*v*) with the bacterial suspension followed by vortexing for 2 min. After 1 h of incubation at 37 °C, the A_600_ value (A) of the aqueous layer was determined and percentage of hydrophobicity was calculated using the Equation (1),

(1) $$\mathrm{Hydrophobicity}\left(\%\right)\;=[(A_{0}-A)/A_{0}]\times 100$$

where A_0_ and A refers to the absorbance values before and after addition of solvent, respectively. The cell surface hydrophobicity of probiotic strain without exposure to different concentrations of oxyresveratrol was maintained as control [22].

### 2.8. Effects on Autoaggregation Potential of the Strain

LF was inoculated in MRS broth for 24 h at 37 °C, and centrifuged (4500× *g*, 10 min, at 20 °C). Cells were washed, resuspended in sterile saline solution (NaCl 0.85 g/100 mL) with different concentrations of oxyresveratrol at (OD_600_) of 0.1. After 1 h of incubation at 37 °C, the absorbance value was measured. For determination of OD_600_ (60 min), the cultures were centrifuged at (300× *g*, 2 min at 20 °C). The percentage of auto aggregation was calculated as Equation (2),

(2) $$\mathrm{Autoaggregation}\;\left(\%\right)\;=\;[\frac{{\mathrm{OD}}_{0}-{\mathrm{OD}}_{60}}{{\mathrm{OD}}_{0}}]\times \;100$$

where OD_0_ is the initial OD value, and OD_60_ refers to the OD value of the suspension after 1 h of incubation. To determine whether oxyresveratrol affects the autoaggregation capacity, probiotic strains without the compound were assayed as control. After 24 h, the cells aggregated were stained with crystal violet and observed microscopically in 100× magnification (Olympus BX51) [6,24].

### 2.9. Effects of Oxyresveratrol on Coaggregation Potential

The test strain, *L. fermentum*, was cultivated at 37 °C for 24 h in MRS broth and harvested through centrifugation at 5000× *g*, 10 min, 4 °C, washed and resuspended in sterile 0.85% NaCl solution. The OD_600_ of the cultures were adjusted to 0.1 and equal volumes of both the test strains and pathogens (*Salmonella enterica* and *Escherichia coli* MDR) were mixed with different concentrations of oxyresveratrol and subjected to incubation at 37 °C for 1 h. The coaggregation capacity was evaluated by measuring OD_600_ at 0 and 60 min of incubation. The percentage co-aggregation was determined using the formula, Equation (3) is as following:

(3) $$\mathrm{Coaggregation}\%=[\frac{{\mathrm{OD}}_{0}-{\mathrm{OD}}_{60}}{{\mathrm{OD}}_{0}}]\times \;100$$

where OD_0_ is the initial OD measured before incubation (0 min) and OD_60_ is the OD measured after 60 min of incubation [25].

### 2.10. Effects of Oxyresveratrol on Antagonistic Activity of the Strain

To evaluate the antagonistic activity of *L. fermentum* against pathogens in the presence of different concentrations of oxyresveratrol, the test strains were grown in MRS broth for 24 h at 37 °C and supplemented with MIC and sub-MIC concentrations of oxyresveratrol. After achieving an OD_600_ of 0.1, 10 µL of each bacterial suspension containing oxy was spotted on MRS agar and incubated for 24 h at 37 °C. Further, 20 µL of the 0.1 OD_600_ adjusted cultures of pathogens (*E. coli* MDR, and *Salmonella enterica* grown in Nutrient broth (HiMedia, India) at 37 °C for 24 h) was mixed with soft agar (0.7% *w*/*v* agar) and overlayed on the spotted MRS agar plates and incubated at 37 °C for 24 h. The plates were checked for growth inhibition zones and the diameters of zones were measured [6,26].

### 2.11. Statistical Analysis

Statistical analysis of data obtained by performing One-way RM ANOVA and Two-way RM ANOVA a with Dunnett’s Multiple Comparison test and Tukey’s multiple comparison tests and values were expressed as mean ± SD (standard deviation of the mean) values of 3 independent experiments using Graph Pad Prism 8.0.2. (GraphPad Software, Inc., San Diego, CA, USA). Significance levels were at * *p* ≤ 0.05, ** *p* ≤ 0.01, *** *p* ≤ 0.001 and **** *p* ≤ 0.0001.

### 2.12. GenBank Accession Numbers

*L. fermentum* ASBT-2 strain is deposited in GenBank with BioSample accession number SAMN15244744. The 16S rRNA sequence of *Salmonella enterica* used in this study was deposited in GenBank with accession number MW116733.

### 2.13. Computational Molecular Docking Studies

The objective of the present computational work was to identify potential efficiency of oxyresveratrol to bind to *Salmonella* effector proteins focusing Salmonella pathogenicity islands SP-I and SP-2. The discovery of binding effectiveness and pharmacokinetic characteristics for phytochemical substances was the main goal. Molecular docking studies aid in the understanding of fundamental biological processes by allowing small molecule interactions with a protein’s active site. By combining the results of computational tools, namely CASTp, ScanProsite, and existing literature references, the binding site of the target protein was found. CASTp considers the area and volume of the pocket while using a solvent accessible surface model and a molecular surface model to locate protein binding site regions [27]. ScanProsite uses context-dependent template annotations, which include biological fingerprints such as expression patterns, to discover structural and functional intradomain residues [28]. FlexX was used to conduct molecular docking. It employs an incremental algorithm that combines a good description of the physicochemical properties with effective methods for sampling the conformational space of the ligand. The technique makes exploring the binding properties of a large number of flexible ligand conformers easier [29]. The determination of the protein active site was followed by the provision of preoptimized 3D coordinates of the ligand. The HYDE scoring function uses the hydrogen bond, torsion energies, and desolvation terms of protein–ligand complexes to estimate binding affinity and ligand efficiency [30].

## 3. Results

### 3.1. Bacterial Strains and Raw Materials

*L. fermentum* ASBT-2 was revived from glycerol stocks and further plated in MRS agar. The coconut shells were coarsely powdered and stored for further extraction.

### 3.2. Extraction and Isolation of Oxyresveratrol

**Figure 1:**
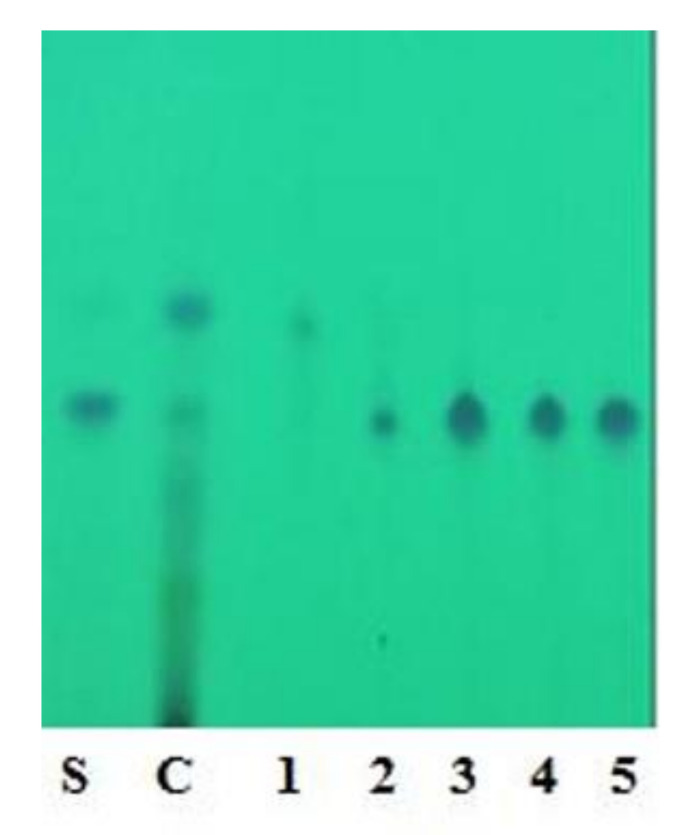
TLC analysis of column fractions. (S: Standard Oxyresveratrol; C: Crude EMK extract; 1–5: fractions collected).

Coconut shell powder (1 kg) after sequential extraction gave a yield of 0.8 g with chloroform and 9 g with EMK extracts. EMK extract showed a corresponding band towards standard oxyresveratrol by TLC. Further purification of EMK extract with column chromatography was performed with different proportions of chloroform and ethyl acetate; the desired band of oxyresveratrol was obtained with 60% (60:40) chloroform elution. Based on the TLC profile, purified fractions with bands corresponding to standard oxyresveratrol were pooled together (Figure 1).

#### Characterization of Oxyresveratrol

**Figure 2:**
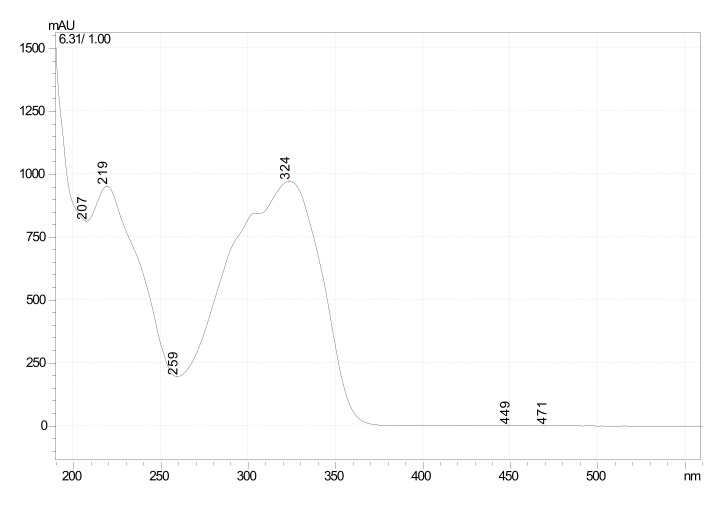
UV-spectrum of oxyresveratrol (UV λmax-219 nm, 324 nm).

**Figure 3:**
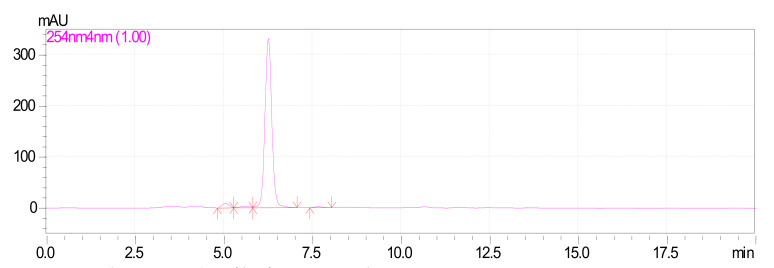
HPLC profile of oxyresveratrol.

Characterization of the desired compound was carried out using further UV, HPLC and LCMS studies. The purified fraction (60% chloroform and 40% ethyl acetate) was obtained as a dull white colored powder which gave pale brown color with FeCl_3_: and readily dissolves in NaOH (1% aqueous), indicating the phenolic nature of the compound. The structural characteristics of F1 are: MP 204 °C–205 °C, UV λmax (MeOH)/nm; 219, 324 (Figure 2) HPLC; Rt-6.2 (Figure 3) matched with standard oxyresveratrol, with 92% purity, LC–MS; [M+1] + (m/e) = 245, MS/MS = 227, 199, 135, 107 (Figure S1). Considering all the data obtained, the purified fraction was characterized as oxyresveratrol (2,3′,4,5′-Tetrahydroxy-trans-stilbene), a naturally occurring polyphenol. The data obtained also matches with the reported data [10].

### 3.3. Determination of Minimum Inhibitory Concentration (MIC) of Oxyresveratrol

**Figure 4:**
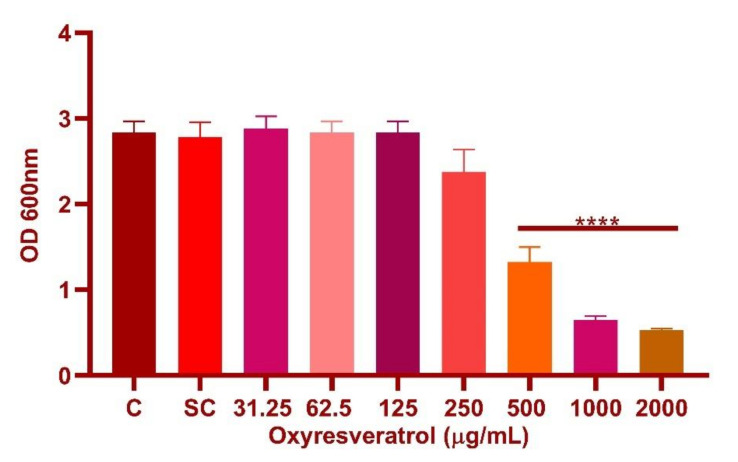
Minimum inhibitory concentration (MIC) of oxyresveratrol against *L. fermentum*. The tested concentrations examined for oxyresveratrol were in the range of 31.25–2000 µg/mL. **** Significantly different (*p* ≤ 0.0001).

The tested concentrations examined for oxyresveratrol were in the range of 31.25–2000 µg/mL (*p* ≤ 0.0001) (Figure 4). Oxyresveratrol showed an MIC of 1000 µg/mL against *L. fermentum* ASBT-2. Further investigations for determining the synergy of the probiotic strains with oxyresveratrol were performed at the MIC and sub-MIC concentrations (MIC/2 and MIC/4) of the compound against the strains.

### 3.4. Effect of Oxyresveratrol on pH Tolerance of the Strain

**Figure 5:**
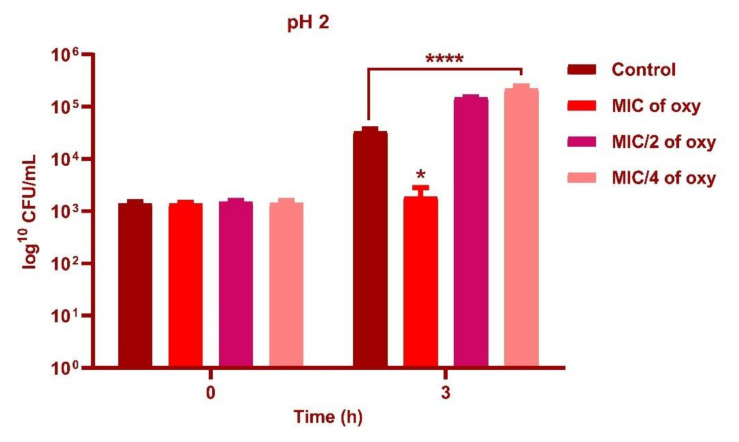
Viable counts (log CFU/mL) of *L. fermentum* ASBT-2 after 3 h of exposure to pH 2.0 with MIC, MIC/2 and MIC/4 concentrations of oxyresveratrol. **** Significantly different (*p* ≤ 0.0001), * Significantly different (*p* ≤ 0.05).

**Figure 6:**
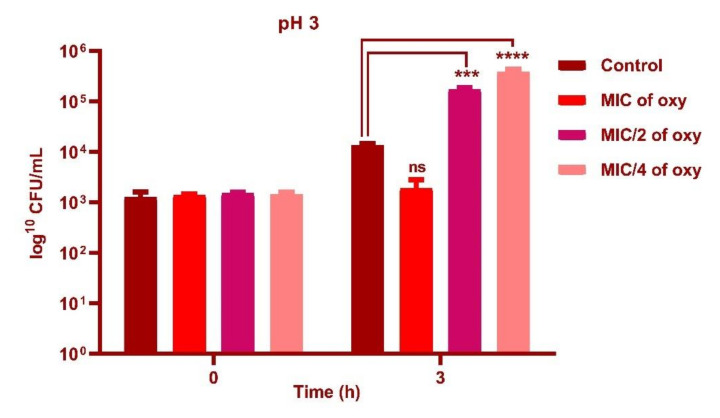
Viable counts (log CFU/mL) of *L. fermentum* ASBT-2 after 3 h of exposure to pH 3.0 with MIC, MIC/2 and MIC/4 concentrations of oxyresveratrol. **** Significantly different (*p* ≤ 0.0001), *** Significantly different *p* ≤ 0.001, ns: non-significant.

**Figure 7:**
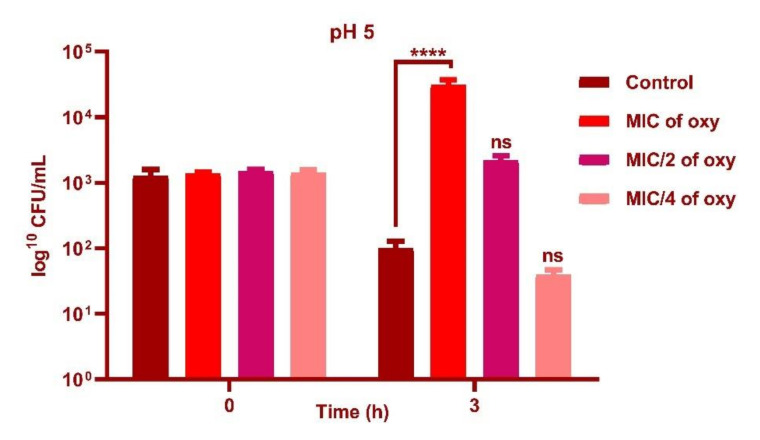
Viable counts (log CFU/mL) of *L. fermentum* ASBT-2 after 3 h of exposure to pH 5.0 with MIC, MIC/2 and MIC/4 concentrations of oxyresveratrol. **** Significantly different (*p* ≤ 0.0001); ns: non-significant.

*L. fermentum* ASBT-2 in sub-MIC concentrations (250 µg/mL) demonstrated an increase in cell counts by 1 log CFU/mL with exposure to pH 2.0 (Figure 5) and 2 log CFU/mL increase when grown in pH 3.0 (*p* ≤ 0.0001) when compared to control (Figure 6). However, at pH 5.0, MIC concentrations (1000 µg/mL) increased viable counts by 2 log CFU/mL (Figure 7). Results substantiated that the addition of the compound could alleviate the tolerance of the probiotic strains to lower pH ranges.

### 3.5. Effect of Oxyresveratrol on Tolerance to Bile Salts

**Figure 8:**
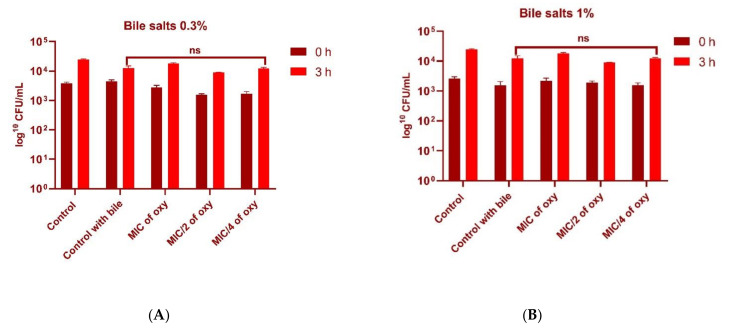
(**A**) Viable counts (log CFU/mL) of *L. fermentum* ASBT-2 after 3 h of exposure to 0.3% bile salts with MIC, MIC/2 and MIC/4 concentrations of oxyresveratrol. (**B**) Viable counts (log CFU/mL) of *L. fermentum* after 3 h of exposure to 1% bile salts with MIC, MIC/2 and MIC/4 concentrations of oxyresveratrol. *L. fermentum* ASBT-2 grown in MRS supplemented with bile salts without compound was maintained as control (control with bile). ns: non-significant.

Bile salts did not significantly increase the tolerance level of the probiotic strain in the presence of the compound. However, both 0.3% and 1% bile salt concentration in the presence of oxyresveratrol did not inhibit the growth of the organism, indicating that tolerance levels of the strain were not altered (Figure 8A,B).

### 3.6. Effects on Survival under Simulated Gastrointestinal Conditions

**Figure 9:**
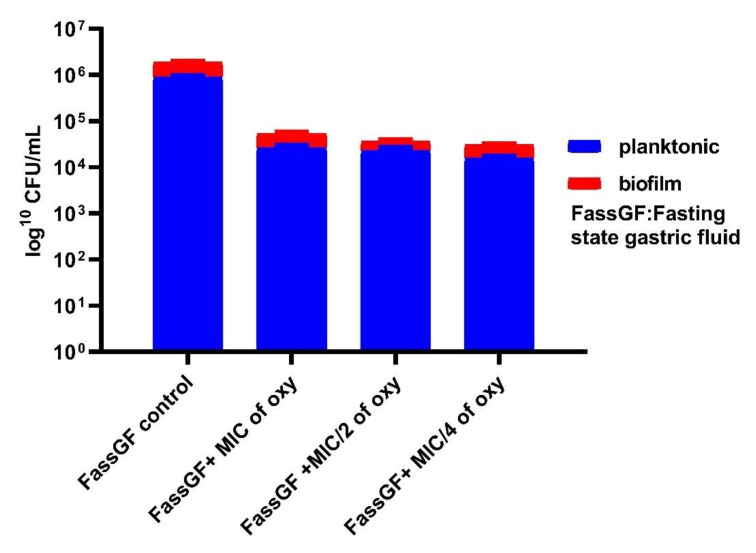
Viable counts (log CFU/mL) of *L. fermentum* ASBT-2 after 3 h of exposure to fasting state gastric fluid (FaSSGF).

**Figure 10:**
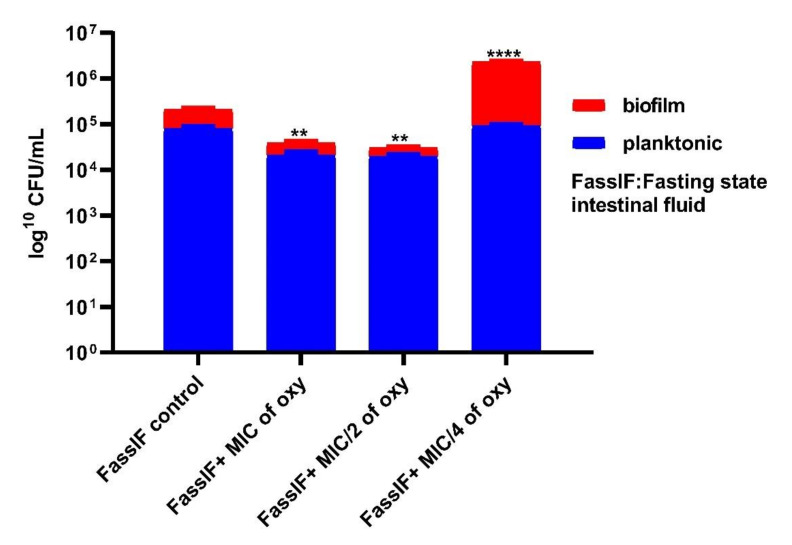
Viable counts (log CFU/mL) of *L. fermentum* ASBT-2 after 3 h of exposure to fasting state intestinal fluid (FaSSIF). **** Significantly different (*p* ≤ 0.0001); ** significantly different (*p* ≤ 0.01).

**Figure 11:**
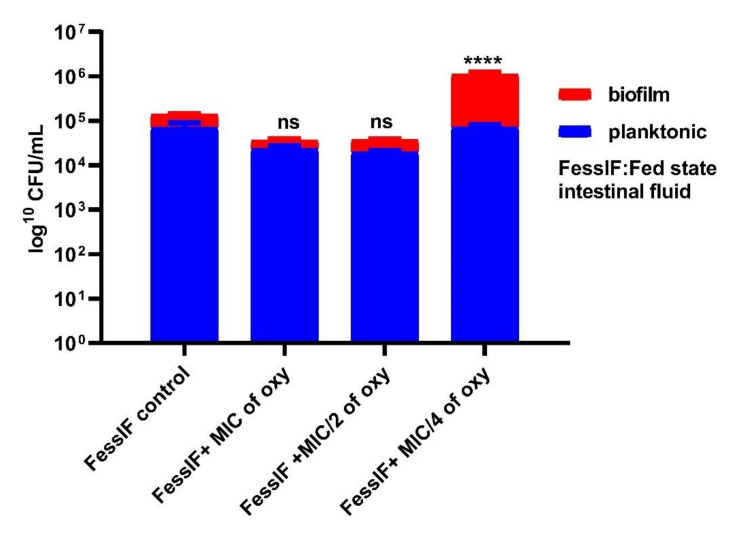
Viable counts (log CFU/mL) of *L. fermentum* ASBT-2 after 3 h of exposure to fed state intestinal fluid (FeSSIF). **** Significantly different (*p* ≤ 0.0001); ns: non-significant.

The ability of the strains to grow in planktonic or biofilm conditions in the gut microenvironment was investigated by simulating the fed and fasting state gastric and intestinal fluids. The viable counts after 3 h of exposure to the different concentrations of the compound indicated that *L. fermentum* ASBT-2 planktonic culture grown in FaSSGF did not alter much in the presence of compound (Figure 9). However, when grown in FaSSIF, biofilm cultures increased by 1 log CFU/mL at MIC/4 (250 µg/mL) concentration of the compound. This would be an essential clue indicating the ability of the strains to adhere to the intestinal epithelium even in a fasting simulated environment (Figure 10). Moreover, in FeSSIF, addition of the compound in MIC/4 concentration demonstrated the same effect, indicating the biofilm formation ability of the strain (Figure 11). Planktonic growth in both fed state and fasting state intestinal fluids was not much altered in the presence of the compound. The results suggest that the biofilm cultures of the probiotic strains could modulate the transit tolerance in the intestine under fasting and fed conditions when compound is incorporated.

### 3.7. Effect of Oxyresveratrol on Cell Surface Hydrophobicity

**Figure 12:**
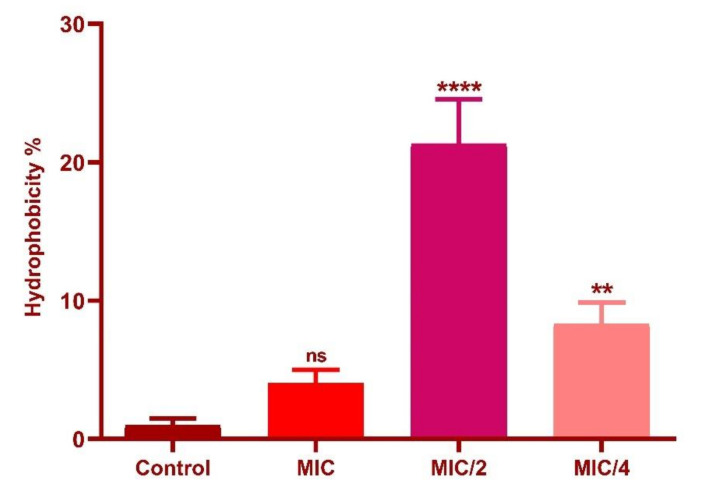
Hydrophobicity percentage of *L. fermentum* ASBT-2 with MIC and sub-MIC concentrations of oxyresveratrol. **** Significantly different (*p* ≤ 0.0001); ** significantly different (*p* ≤ 0.01); ns: non-significant.

MATH assay was performed on n-hexadecane as the solvent and a significant increase in cell surface hydrophobicity of *L. fermentum* was induced at MIC/2 (*p* ≤ 0.0001) and MIC/4 (*p* ≤ 0.01) of the compound (Figure 12) when compared to control. The observations could provide evidence to the ability of the compound to promote the adherence ability of the strains to the hydrophobic barriers in the gut epithelium.

### 3.8. Effects on Autoaggregation Potential of the Strain

**Figure 13:**
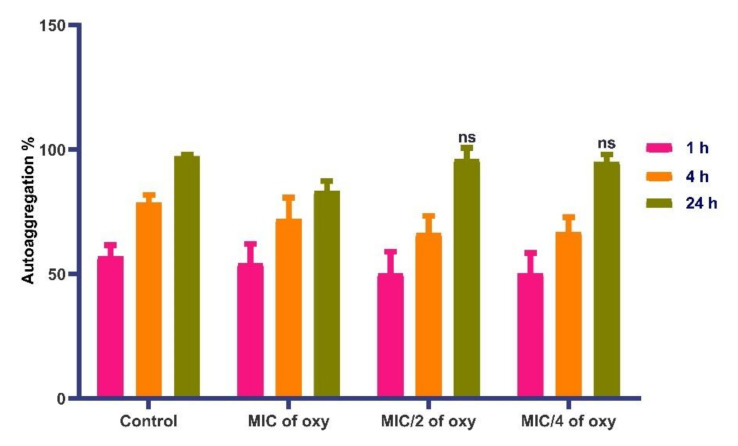
Autoaggregation potential of *L. fermentum* ASBT-2 with MIC and sub-MIC concentrations of oxyresveratrol at 1 h, 4h and 24 h of incubation. ns: non-significant.

**Figure 14:**
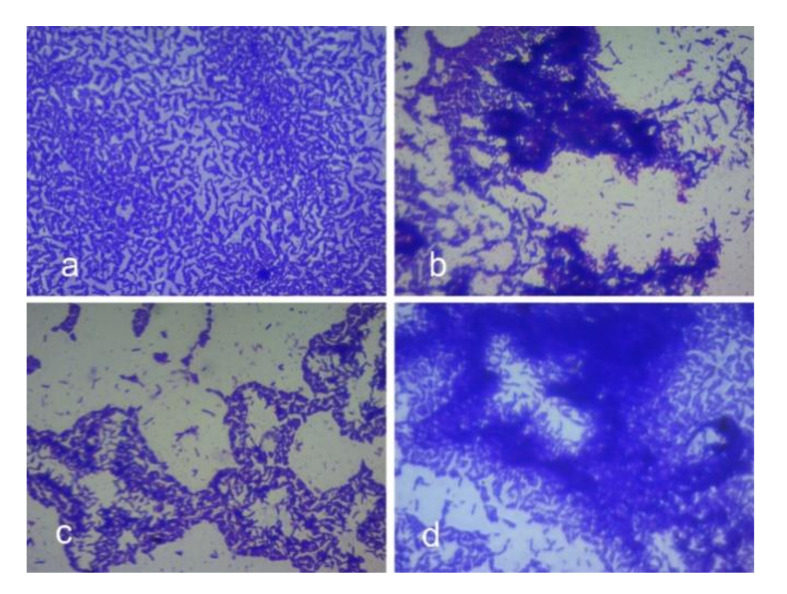
Microscopic images (crystal violet stained) of *L. fermentum* ASBT-2 demonstrating autoaggregation when treated with different concentrations of oxyresveratrol after 24 h (100× magnification). (**a**) Control without oxyresveratrol; (**b**) cells treated with MIC of oxyresveratrol; (**c**) cells treated with MIC/2 of oxyresveratrol; (**d**) cells treated with MIC/4 of oxyresveratrol.

*L. fermentum* autoaggregated in the range of 94% in sub-MIC concentrations of the compound with similar traits as the control (Figure 13). In MIC concentration, autoaggregation was in the range of 79%. The results suggested that autoaggregation efficiency remains unaltered with the addition of the compound, indicating the effectiveness of the combination in lower doses. These observations were further confirmed by the microscopic images (crystal violet stained) images of *L. fermentum* (Figure 14a–d).

### 3.9. Effects of Oxyresveratrol on Coaggregation Potential

**Figure 15:**
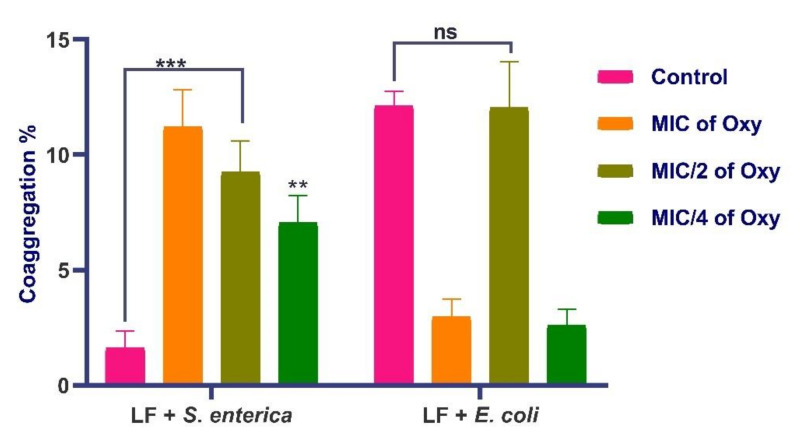
Coaggregation potential of *L. fermentum* ASBT-2 (LF) against *S. enterica* and *E. coli* MDR with different concentrations of oxyresveratrol. *** Significantly different (*p* ≤ 0.001); ** significantly different (*p* ≤ 0.01); ns: non-significant.

*L. fermentum* ASBT-2 at all concentrations of MIC of the compound significantly coaggregated with *S. enterica* (Figure 15). With *E. coli* MDR, the *L. fermentum* strain coaggregated with same efficacy as the control when treated with MIC/2 concentration of oxyresveratrol. Effective coaggregation will significantly enhance the ability of the probiotic strains to exclude pathogenic strains from the gut epithelium. Further, the antagonistic potential of the combination against the same pathogens was checked.

### 3.10. Effects of Oxyresveratrol on Antagonistic Activity of the Strain

**Figure 16:**
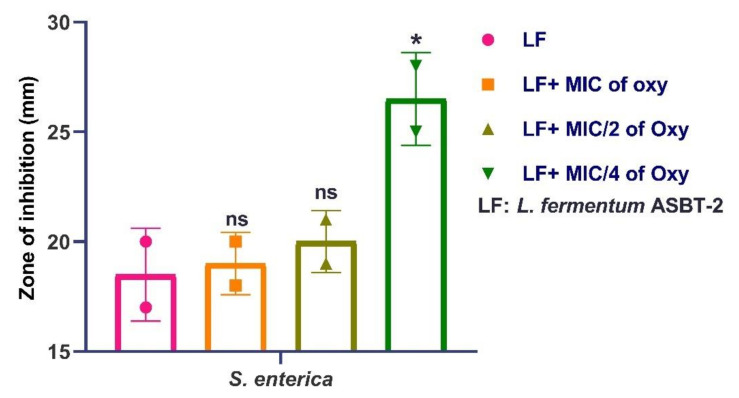
Antagonistic potential of *L. fermentum* ASBT-2 inhibiting *S. enterica* with different concentrations of oxyresveratrol. * Significantly different (*p* ≤ 0.05); ns: non-significant.

Results demonstrated that MIC/4 concentration of the compound significantly inhibited *S. enterica* as demonstrated by the increase in the inhibition zones (Figure S2; Figure 16). Further confirmations on the mechanism of oxyresveratrol inhibiting the pathogen in sub-MIC concentrations was proposed through in silico docking approaches.

### 3.11. Molecular Docking Study Interactions

**Figure 17:**
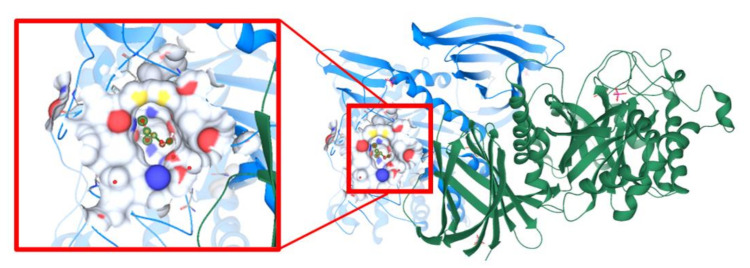
Protein crystal structure of SrfJ from *Salmonella* *typhimurium* with PDB ID: 2WNW. Enlarged region represents the binding site of the protein Chain A with cocrystal glycerol.

**Figure 18:**
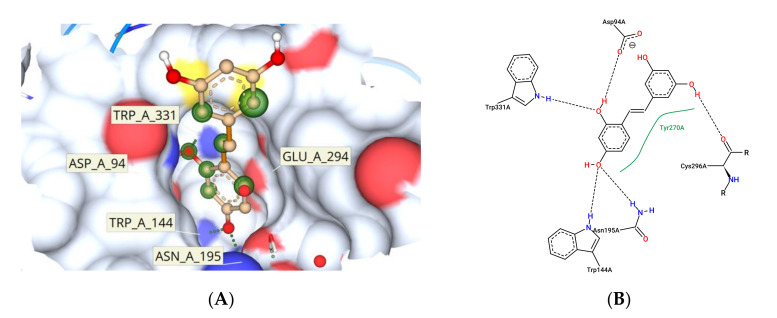
Oxyresveratrol bound to SrfJ protein. (**A**) Three-dimensional depiction of the interaction with key amino acids. (**B**) Two-dimensional depiction of the protein–ligand interaction highlighting the amino acids with hydrogen bonds as well as hydrophobic interactions.

**Figure 19:**
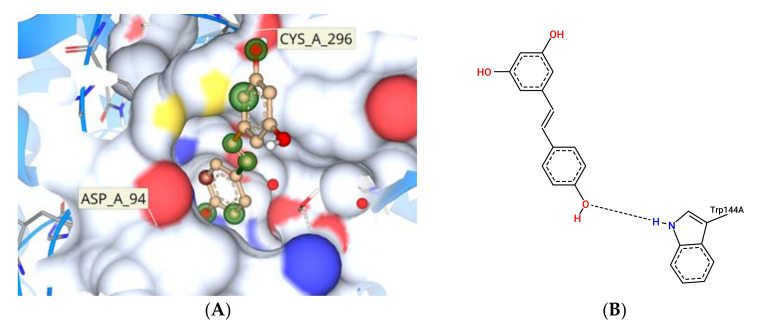
Resveratrol bound to SrfJ protein. (**A**) Three-dimensional depiction of the interaction very limited key amino acids. (**B**) Two-dimensional depiction of the protein–ligand interaction highlighting the amino acids with hydrogen bond interactions.

Molecular docking studies aid in understanding of the protein–ligand interactions. SrfJ from *Salmonella* *typhimurium* was the target protein (PDB ID:2WNW) [31], after screening potential target effector proteins specific to *Salmonella enterica* (Figure 17). Computational techniques such as CASTp and ScanProsite were used to identify active site residues. The active site residues discovered in these two sources were matched to data on SrfJ inhibitors in the literature. ARG87, CYS93, ASP94, PHE95, TRP144, ASN195, GLU196, LYS201, TRP203, ASP204, ASP239, HIS268, TYR270, GLU294, CYS296, TRP331, ASN332, ASN342, ASN346, CYS348, and SER446 were identified as binding site residues (Figure 18A). Tyr276 represented the hydrophobic residues found in the N-terminal lobe of SrfJ among the other binding site residues (Figure 18B). FlexX molecular docking experiments enabled various stereo conformations of ligand molecules such as E/Z, R/S, and pseudo R/S. Per iteration, 60 poses were created, including fragmentation, and the best docking poses were evaluated based on the binding affinity range, ligand efficiency, and torsion parameters. The interactions were compared with binding of resveratrol to SrfJ (Figure 19A,B). Oxyresveratrol was found to be a better candidate in terms of binding affinity to SrfJ and ligand efficiency when compared to resveratrol (Table 1).

## 4. Discussion

The human gastrointestinal (GI) tract represents one of the largest interfaces between the host and environment, harboring trillions of microorganisms [32]. Polyphenols, after conversion to simpler metabolites, have been shown to influence the activities of gut microbiota such as enhancing the colonization of beneficial microorganisms, thereby making them a potential prebiotic [33,34]. Microbial dysbiosis has always been symbolized by an imbalance in gut microbiota leading to disease conditions in neonates and adults [32]. Probiotics and prebiotics are safer dispositions in restoring the gut imbalance as evidenced by multitudes of investigations [34,35]. Many studies have focused on approaches where dietary phenolics have been investigated for their metabolic effects on the gut microbiome [36]. Hernández et al. observed that phenolic extracts of grape pomace have no inhibitory effects on the growth of *L. acidophilus* [37], and another investigation proved that resveratrol, a polyphenol, has antibacterial and anti-adhesive attributes which makes it a potential therapeutic for a periodontal disease caused by *Porphyromonas gingivalis* [38]. Similar observations have also been reported by O’Connor et al. earlier [39].

Underutilized agro-waste products were used for the extraction of a stilbene phytochemical, oxyresveratrol, and the synergy of the compound with probiotic strain *L. fermentum* ASBT-2 isolated in our previous study was explored. The strain possessed potential probiotic properties, and a synbiotic approach was used, where the combination with polyphenolic compound may increase their efficiency as a protective culture application. The potential synergy between probiotics and polyphenols provides a strategy to enhance gut health. Polyphenols can be metabolized by the gut microbiota and the metabolites produced can cause fluctuations in the intestinal microbial composition as investigated by Tugba et al. [40]. Studies have proven the persistence of ingested polyphenolics in the intestine due to their poor absorption and thereby are available in excess to the gut microbiome. The effect and their mechanisms could be manyfold, but very often are cited for their synergy and protective effects on the residual beneficial microbiota [40,41]. Hence, the combination of such polyphenolics with probiotics can be a strategy to enhance probiotic effects, thereby enhancing the host’s health. Our approach of delivering oxy with probiotic strain may open a novel approach to address these issues.

Tolerance to the harsh conditions of GIT, deconjugation of bile salts, cell surface hydrophobicity, adherence to intestinal cells, ability to autoaggregate and coaggregate to pathogenic bacterial strains and antagonistic activity towards pathogens are some of the properties that determine the survival and functioning of probiotic bacteria [42]. Oxyresveratrol demonstrated high MIC values which was indicative of low inhibitory effects on target strains. However, the sub-MIC concentrations (500 µg/mL and 250 µg/mL) of the compound was used to evaluate the efficiency of the combinations. Probiotics that are administered orally must overcome the stressful conditions in the host gastrointestinal tract. Thus, tolerance to low pH and bile salts are important characteristics of probiotic strains in order to survive and colonize the gastrointestinal tract [42]. Our study showed that at low pH values (2.0 and 3.0) LF increased in counts with sub-MIC concentrations of the compound. Tolerance to bile salts being a prerequisite to confirm probiotic attributes, the combinations ensured that at lower concentrations of the compound, bile tolerance ability was not altered. Probiotics *Lactobacilli* and *Bifidobacteria* have adapted efflux pump mechanisms and bile salt hydrolase mediated mechanisms to exclude bile salts [43]. Polyphenols’ ability to alter gut biota to regulate the bile acid pool have been investigated in conditions of coronary heart diseases [44]. These observations may lead to proposing a polyphenol–probiotic synbiotic approach, but future mechanism-based studies could potentially shed light on further applications.

The stomach pH varies between 1.0 and 2.0 with secretion of HCl and the digestive enzyme pepsin while the pH of the small intestine falls between 5.1 and 7.5 with several digestive enzymes [42]. Biofilm and planktonic growth of the probiotic strains is crucial in the gastrointestinal tract (GIT) for balancing the interim microbiota and to exclude the pathogen entry. Here, a different approach was attempted where the simulated gastric and intestinal environment was created with biorelevant dissolution media such as FaSSGF, FeSSIF and FaSSIF. These media contain natural surfactants such as bile salts and phospholipids present in the gut with change in pH of the medium to simulate gastrointestinal fluids [23]. Studies by Mountzouris et al. have proven the effects of probiotic microflora in fasted and fed state mice models [45]. They showed that addition of *L. acidophilus* significantly restored the crucial metabolic enzyme activities in the gut and increased volatile fatty acids (VFA), a prerequisite for colon health. These investigations shed light on the approach of incorporating probiotic strains in combination with the compound in fasting and fed state intestinal juices. FaSSIF and FeSSIF with sub-MIC combinations of the compound restored the biofilm formation and thereby may favorably influence the transit tolerance ability of the strains. It was noteworthy that planktonic populations and biofilm formation of *L. fermentum* was not much altered in fasting state gastric fluids in combination with the compound, indicating that the tolerance regime is not much altered in the gastric environment of the stomach.

Capability to adhere to the host intestinal epithelial cells is a vital property of probiotic bacteria as it is necessary for both initial colonization and protection against pathogenic bacteria via competition for host cell binding sites [46]. Adhesion to intestinal cells of the host also helps in the antagonistic activity against pathogens through the production of antimicrobial compounds and in immunomodulation. Both hydrophobicity and interactions with host cell proteins are known to influence this binding [46]. *L. fermentum* showed an increase in cell surface hydrophobicity with sub-MIC concentrations of the compound. It was evident that the compound enhanced the cell surface hydrophobicity of the strain and hence is likely to enhance host binding. This was in agreement with a study carried out by Santos et al. [6] where an increase in the cell surface hydrophobicity of four *lactic acid bacteria* was observed in the presence of different concentrations of quercetin. Similarly, Volstatova et al. reported an increase in the adhesion of the strains *L. gasseri* R and *L. casei* FMP to Caco-2 cells in the presence of apple peel extract [47]. The flavan-3-ols were also found to have an influence on the adhesion of lactobacilli strains to the intestinal epithelial cells as investigated by Bustos et al. [48]. The ability to aggregate is an important feature of probiotics in order to restore biofilm formation, intestinal mucosal adhesion and protection from the host immune system [49,50]. The addition of the compound maintained the autoaggregation properties of the strain which would be a significant survival advantage for the strain to prevent competitive exclusion. Similarly, higher coaggregation percentages of LF against *S. enterica* and the ability of the strain to effectively inhibit *S. enterica* in the presence of the compound adds strength to the protective effects. Oxyresveratrol has been reported to inhibit Gram positive and Gram negative bacteria in several investigations [51]. The compound was also reported to inhibit uropathogenic *E. coli* biofilms [52] and inhibition of quorum sensing in *Chromobacterium violaceum* [53]. Oxy is also reported to exhibit synergy with the antibiotics ciprofloxacin and gentamicin by permeabilizing the bacterial cell membrane [54]. Fewer investigations are available for proving antibacterial activity of oxy against *Salmonella enterica* and hence could be predicted that since they exhibited rapid absorption into the gastrointestinal tract in previous studies [55], their synergy with the probiotic strain will increase the protective effects. To confirm the possible targets of virulence factors which may be downregulated by the compound, in silico docking approaches was performed with the specific target of effector proteins in the *Salmonella* pathogenicity island SPI and SP2 with oxy. In earlier studies Yang et al. utilized molecular docking and proteome analysis to identify the mechanism of action of pterostilbene which was shown to possess biofilm reduction potential and antimicrobial activity against MRSA [56].

SrfJ is a specific effector located in SPI-2 encoded secretion [57]. Studies by Julia et al. have provided evidence for downregulation of expression of SrfJ leading to reduced proliferation inside host macrophages [58]. Hence, attempts to analyze the specific binding affinity of oxyresveratrol to residues on SrfJ modelled crystal structure of *Salmonella enterica* strain used in the study was performed. Earlier modelling in silico approaches have proved that SrfJ expressed in *Salmonella* *typhimurium* demonstrated greater amino acid sequence homology with human lysosomal glucosylceramidase (GlcCerase) [31]. SrfJ GlcCerase activity has been predicted to enhance *Salmonella* virulence in the earlier study and hence the efficiency of oxyresveratrol to bind to these specific sites compared with binding affinity to resveratrol was investigated. Resveratrol has been reported to downregulate viability of *S.* *typhimurium* induced nitric oxide production thereby implicating its application as a potential drug lead candidate [4]. Potential target proteins such as ST4351 were inhibited by the compound as per Kores et al. [59]. However, when *S. enterica* was modelled with SrfJ as a potential target, oxyresveratrol was found to be a better candidate in terms of binding efficiency to the effector protein. Further investigations need to be performed to understand how the virulence regime of the strain is reduced by addition of the compound with special reference to the binding affinity to SrfJ. SrfJ being one of the less studied SP-2 effector proteins of *Salmonella* in terms of interaction partners and functions, more investigations to study the interaction of oxyresveratrol to SrfJ key residues by in vitro approaches can be a future direction.

## 5. Conclusions

A potential polyphenolic stilbene compound, oxyresveratrol was successfully isolated, and characterized from underutilized agro-waste. Survival of probiotic strain *L. fermentum* ASBT-2 in the challenging environment of GIT and enhancement of their inherent probiotic properties was effectively complemented with the addition of sub-inhibitory concentrations of the compound. This could explore a promising approach to increase the gut barrier protection with potential complementation of underutilized compounds as dietary supplements. Oxy–probiotic synergy in sub-MIC concentrations inhibited growth of the enteric pathogen, *Salmonella enterica*. In silico molecular docking approaches confirmed the interaction of oxy with SrfJ effector proteins of *Salmonella* spp. This could indicate a strong cue for the interaction of oxy with SPI-2 effector proteins of the pathogen targeting the virulence property of the strain. Future approaches to understand the gut barrier modulation by addition of stilbene compounds should be performed which would shed light on improvement of host defense mechanisms.

## Figures and Tables

**Table 1:** Molecular docking parameters for 2 ligands against the protein 2WNW. Binding affinity is represented as a range as it cannot be predicted as an absolute value accurately through in silico methods. Torsion bonds were statistically compared with Cambridge Structural Database to understand the co-crystal formation of the compound docked against the protein. Inter- and intra-ligand clashes were analyzed and reported.

Sl.No.	Compound	Binding Affinity	Ligand Efficiency	LogP	Torsions	Clashes
1	Oxyresveratrol	μM-mM	Acceptable	2.97	Matches with CSD	No Clashes
2	Resveratrol	Towards mM	Not Acceptable	2.05	Matches with CSD	Inter Clashes

## Data Availability

Not applicable.

## Supplementary Materials

The following are available online at https://www.mdpi.com/article/10.3390/foods10112548/s1, Figure S1: MS and MS/MS data of oxyresveratrol, Figure S2: Agar spot assay indicating inhibition of *S. enterica* overlayed with different concentrations of oxyresveratrol (MIC, MIC/2 and MIC/4) with *L. fermentum* ASBT-2. Control was culture of *L. fermentum* spotted without compound.
