# Supplementary material for: Synergistic Effects of Limosilactobacillus fermentum ASBT-2 with Oxyresveratrol Isolated from Coconut Shell Waste
Source: Foods. 2021 Oct 22;10(11):2548. doi: 10.3390/foods10112548 (PMC8622123; doi:10.3390/foods10112548)
Supplement: Supplementary file 1 [file foods-10-02548-s001.zip › foods-1396829-supplementary.pdf]

Supplementary Material

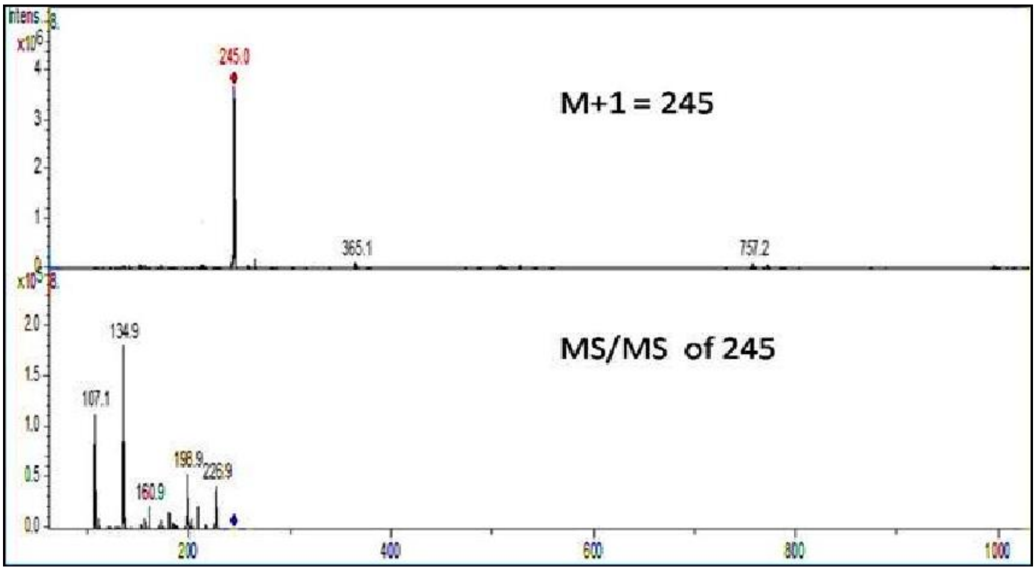

Figure S1. MS and MS/MS data of oxyresveratrol

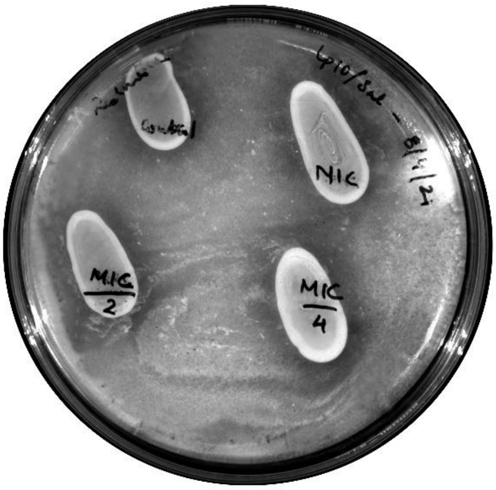

Figure S2. Agar spot assay indicating inhibition of *S. enterica* overlayed with different concentrations of oxyresveratrol (MIC, MIC/2 and MIC/4) with *L. fermentum* ASBT-2. Control was culture of *L fermentum* spotted without compound.
